# Taify Pomegranate Juice (TPJ) Abrogates Acrylamide-Induced Oxidative Stress Through the Regulation of Antioxidant Activity, Inflammation, and Apoptosis-Associated Genes

**DOI:** 10.3389/fvets.2022.833605

**Published:** 2022-03-22

**Authors:** Ahmed M. El-Shehawi, Samy Sayed, Mohamed M. Hassan, Saad Al-Otaibi, Fayez Althobaiti, Mona M. Elseehy, Mohamed Soliman

**Affiliations:** ^1^Department of Biotechnology, College of Science, Taif University, Taif, Saudi Arabia; ^2^Department of Science and Technology, University College-Ranyah, Taif University, Taif, Saudi Arabia; ^3^Department of Biology, College of Science, Taif University, Taif, Saudi Arabia; ^4^Department of Genetics, Faculty of Agriculture, University of Alexandria, Alexandria, Egypt; ^5^Clinical Laboratory Sciences Department, Turabah University College, Taif University, Taif, Saudi Arabia

**Keywords:** acrylamide (Acr), pomegranate juice, antioxidant, liver dysfunction, oxidative stress

## Abstract

Acrylamide (ACR) has various effects on biological systems, including oxidative stress and its associated metabolic disorders. Previous research reports that plants growing at high altitude have a different profile of antioxidants. In the current report, the Taify pomegranate juice (TPJ) of the Taify pomegranate growing at the Taif region (high altitude), Saudi Arabia, was investigated for its protective activity from ACR-induced oxidative stress. Rats were treated with ACR, TPJ, or TPJ+ACR, and various assays, including blood chemistry, liver function biomarkers, gene expression of endogenous antioxidant enzymes, oxidative stress regulatory genes, inflammation biomarkers, and apoptosis, were estimated using biochemical, real-time PCR, histopathological, and immunohistochemical analysis. TPJ showed a protective function of ACR-induced alteration of AST, ALT, GGT, urea, total proteins, albumin, MDA, and NO. It also increased the level of the endogenous antioxidative enzymes, including SOD, catalase, and GSH. It showed anti-inflammatory activity by reduction the TNF-α, IL-6 secretion and the enhancing of IL-10 levels. At the gene expression level, TPJ upregulated the expression of endogenous antioxidant genes (SOD and catalase) and of antioxidant-regulating genes Nrf2 and HO-1; downregulated the expression of inflammatory genes TGF-β1, COX2, and the apoptotic gene caspase-3; and upregulated the expression of antiapoptotic gene Bcl2. At the histological level, TPJ showed a protective effect from the ACR-induced hepatic histological damage. Results of this study conclude that TPJ has a protective effect from ACR-induced oxidative stress and its associated metabolic alterations through its antioxidant and anti-inflammatory activities.

## Introduction

Acrylamide (ACR) represents a major threat for human health. It is considered to be one of the most important chemical materials that is widely used in industry. It is also one of the inevitable contaminants in the occupational environment (1). Besides this, it is detected in the cooked starchy foods (1, 2). It was declared a carcinogenic agent in 1994 by The International Agency for Research on Cancer (3). Various studies report the wide range of ACR's negative impact on health, including oxidative stress and cancer (1, 4–8), neurotoxicity (1, 3, 4, 9, 10), reproductive toxicity (1, 4, 11), and developmental toxicity (1, 12). Acrylamide exerts its harmful effects by increasing the level of reactive oxygen species (ROS) and decreasing the antioxidant capacity via its deleterious effects to the endogenous antioxidant enzymes, including superoxide dismutase (SOD) and glutathione peroxidase (GSH-Px) (13, 14). Also, it provokes the production of inflammatory cytokines, including TNF-α and IL-1β (12, 14), and induces mitochondrial and caspase-dependent apoptosis (15, 16).

During the last two decades, the use of herbal therapy in counteracting the negative impact of oxidative stress, pollution, obesity, aging, and tissue deterioration and synthetic drugs as well as their associated metabolic disorders has gained a lot of research interest worldwide (17–21). Various terms were coined for natural products that are used in herbal therapy, including functional foods, food supplements, or nutraceuticals (17, 21). They are used for treatment of metabolic disorders associated with oxidative stress without having the side effects of chemically synthesized drugs (20). Their antioxidant activity is due to the presence of various phytochemicals (22), mainly including flavonoids and phenolic compounds (23–25). They are detected in a wide range of plant species, including cereals, fruits, vegetables, and oil seeds (19, 21, 26–29).

A plethora of investigations has been published reporting different strategies to combat ACR toxicity and its associated deleterious effects, especially the use of plant-driven phytochemicals (15). Among these natural compounds are thermoquinone and capsaicin (30), N-acetylcysteine (31–34), quercetin (35–37), punicalagin (38), vitamin E (39), carboxyflurene (40), cyaniding-3-O-glucoside (41), and thymoquinone (8, 42). Similarly, plant extracts are used to counteract the impact of ACR, including *Trigonella foenum*-graecum seed oil (43); *Spirulina platensis* (44); *Tetrastigma hemsleyanum* leaf extract (45); *Portulaca oleracea* seed extract (46); white tea and raspberry ketone (47); hesperidin and tiger nut (48); olive oil hydroxytyrosol (49); tea polyphenols and resveratrol (50); and strawberry, grape, and blueberry powder (51).

Chemical constituents of plant extracts and essential oil differ based on geographical locations, altitude, and genotypes. Samples collected from 20 different geographical locations of *Ducrosia anethifolia* showed variations in essential oil (52). Chemical constituents of pomegranate fruits vary with climate conditions (53). Phytochemical variations among genotypes are also reported in tomato (54). Metabolite analysis of *Curcuma longa* shows differences up to 20% due to different agroclimatic regions (55).

Plants respond to high altitude by producing higher levels of a wide array of antioxidants compared with plants growing at lower altitude (56, 57) or producing *de novo* compounds not detected at normal elevations (58, 59). For example, a significant increase in total phenolics and antioxidant activity was reported in the methanolic extract of *Scrophularia striata* growing at an elevation of 600 m above sea level (60). Similarly, high altitude is negatively correlated with the content of tannin and positively correlated with flavonoids, rutin, total phenolic content, and antioxidant capacity of *Potentilla fruticosa L*. extract (61). In addition, phytochemical activity of *Thalictrum foliolosum* root extract collected from different altitudes shows that berberine content was negatively correlated with altitude while total phenolics and flavonoid as well as antioxidant capacity were positively correlated with altitude (62). The phenolic content and antioxidant capacity of two major *Rhodiola* species differed in samples collected from different elevations based on the differential abundance of 178 flavonoids, which positively correlated with elevation (63). The phenol and flavonoid contents *Thalictrum foliolosum* as well as antioxidant capacity increased at higher altitudes (62). Therefore, phytochemical content of plants depends on various factors, especially their elevation above sea level.

Pomegranate juice (PJ) shows a wide spectrum of effects, including antioxidant activity (64–75), antimutagenic activity (65), antiviral activity (76), antiobesity (19, 77), anticryptosporidial (78), and amelioration of neurodegenerative diseases (79). Also, pomegranate peel shows antioxidant (64, 68, 80, 81) and antimicrobial activity (82). In addition, several reports document that pomegranate seed extract presents antioxidant activity (64, 68, 83, 84).

Taif is a high-altitude region elevated about 1,200–2,300 m above sea level, which makes it suitable for a wide variety of indigenous plant species (85). This makes it unique in weather and plant flora. Among the most important Taif indigenous fruit plants are grape and pomegranate, *Punica granatum L*. (Lythraceae). The most cultivated variety of pomegranate is known as Taify, which has special cultural, nutritional, and commercial value in the Kingdom of Saudi Arabia (19). The elevated location of Taif as an indigenous habitat for Taify pomegranate makes this variety a potential special antioxidant source.

The previous theoretical framework detailed that few studies have been conducted to investigate the different aspects of Taify pomegranate (19, 86), which does not correlate with its economic, nutritional, and cultural value. Therefore, the focus of the current study was to investigate the antioxidant activity of Taify pomegranate juice (TPJ) against ACR-induced oxidative stress on the liver of rats. Various assays including biochemical, molecular, inflammatory, apoptotic, and histopathological were employed to achieve this goal.

## Materials and Methods

### Plant Samples and Extraction

Taify pomegranate fruits were collected from their natural habitat in the Taif region, Taif Governorate, Saudi Arabia. Intact seeds were collected from healthy ripened fruits, and TPJ was extracted by hand squeezing in cheesecloth without disrupting seed structure to get only the juice components. TPJ was filtered using a 0.22-μm filter and administered fresh to rats.

### Animals and Treatments

Forty male rats (Ratus norvegicus) 10 weeks of age were used in this study. Animals were cared for at room temperature with free access to food and water for 7 days for acclimation at Turabah University labs. Animals were randomly and equally distributed among four groups. Group 1 served as the negative control and was given an equal volume of water. Group 2 orally received 20 mg/kg bw of acrylamide and served as the positive control (ACR group) (87, 88). This was reported to induce total liver toxicity (89). Group 3 (TPJ) orally received 2 ml/Kg bw of TPJ for 3 weeks (90). Group 4 (TPJ+ACR), the protective group, received 20 mg/kg bw of ACR and 2 ml/kg bw of pomegranate juice. TPJ administration started 1 week earlier than ACR administration for the protective effect. Treatments continued for 3 weeks. After the experimental period, animals were anesthetized using isoflurane, decapitated, and dissected. Blood samples were collected, and serum was separated and stored at −20°C for blood chemistry assays. Liver tissue samples were collected in QIAZOL reagent for RNA isolation and quantitative real-time PCR (qRT-PCR) analysis of gene expression. Liver samples were kept in Bowan's solution for histological and immunohistochemical analysis.

### Blood Biochemical Analysis

The serum levels of ALT, AST, GGT, and urea were measured using a colorimetric spectrophotometer as described in the instruction manual. Malondialdehyde (MDA) was measured according to a previously described method (91). Catalase, superoxide dismutase (SOD), and nitric oxide (NO) were estimated according to previously reported methods (92, 93). Well-established methods were employed to measure albumin (94) and total proteins (95). GSH was estimated according to the Tietze method (96) using spectrophotometric absorbance at 412 nm.

### Analysis of Inflammation and Anti-inflammation Cytokines

IL-6 and TNF-α were estimated using specific ELISA (ab100768 and ab46070, respectively) kits and spectrophotometric analysis according to the kits' instructions. IL-10 was measured using a commercial kit obtained from Abcam, USA (Rat IL-10 ELISA Kit, ab100765). Data obtained from the ELISA reader were calculated as described in the kit instructions.

### Gene Expression Quantitation by Real-Time PCR

Total RNA was isolated from liver samples using QIAZOL according to the manufacturer's instructions, 50 μg per 1 ml of QIAZOL. Concentration of the isolated RNA was estimated spectrophotometrically (BIORAD, USA) at 260 nm. Total RNA, 2 μg, was used as a template for reverse transcriptase for cDNA synthesis (MyTaq Red Mix, Bioline). cDNA was used as a template for qPCR amplification of various liver using SYBR Green master mix (Thermo scientific, USA). Primers were synthesized at Macrogen Company (Seoul, Korea). Information of primers used in qPCR amplification are summarized in Table 1. The obtained qPCR data was analyzed using the 2^−ΔΔ^Ct method of the CFX96 Touch Real-Time PCR (Bio-Rad, USA). Beta-actin gene expression was used as a reference for estimation of gene expression.

**Table 1:** Summary of primer information used for quantitative real-time PCR in rat liver.

**Gene**	**Primer name**	**Sequence 5^**′**^-3^**′**^**	**Accession number**
Bcl2	Bcl2-F	ACTCTTCAGGGATGGGGTGA	NM_016993
	Bcl2-R	TGACATCTCCCTGTTGACGC	
HO-1	HO-1-F	GTAAATGCAGTGTTGGCCCC	NM_012580.2
	HO-1-R	ATGTGCCAGGCATCTCCTTC	
BAX	BAX-F	AGGACGCATCCACCAAGAAG	NM 017059
	BAX-R	CAGTTGAAGTTGCCGTCTGC	
Nrf2	Nrf2-F	TTGTAGATGACCATGAGTCGC	NM_031789.2
	Nrf2-R	TGTCCTGCTGTATGCTGCTT	
TGF-β1	TGF-β1-F	GGACTACTACGCCAAAGAAG	NM_021578.2
	TGF-β1-R	TCAAAAGACAGCCACTCAGG	
COX2	COX2-F	TGATCTACCCTCCCCACGTC	NM 017232
	COX2-R	ACACACTCTGTTGTGCTCCC	
SOD	SOD-F	ACACCTATGCACTCCACAGAC	NM_053425.1
	SOD-R	ACATTCGACCTCTGGGGGTA	
Catalase	CAT-F	GCGGGAACCCAATAGGAGAT	NM_012520.2
	CAT-R	CAGGTTAGGTGTGAGGGACA	
β-actin	β-actin-F	AGGAGTACGATGAGTCCGGC	NM 031144
	β-actin-R	CGCAGCTCAGTAACAGTCCG	

### Histopathology and Immunohistochemistry

Liver tissue samples were fixed in neutral buffered formalin (10%). Fixed samples were processed and stained with hematoxylin and eosin as described by Bancroft et al. (97). The immunohistochemical protocol was conducted following the method of Saber et al. (98). Wax was removed from tissue sections, and they were rinsed in 0.05 M citrate buffer, pH 6.8. Non-specific binding was blocked by treating the sections with 0.3% H_2_O_2_ and a protein block. Sections were subjected to rabbit monoclonal (anti-bcl2, Abcam, Cat# ab182858, dilution 1:500) primary antibody. Sections were washed in phosphate-buffered saline and subjected to goat antirabbit secondary antibody (EnVision System Horseradish Peroxidase Labeled Polymer; Dako) for 30 min at room temperature. Slides were visualized with DAB kit and counterstained using Mayer's hematoxylin. The immunolabeling indices of Bcl2 are presented as a percentage of positive expression in a total of 1,000 cells per eight high power fields (HPF).

### Statistical Analysis

The SPSS program (IBM, Chicago, IL, USA) was used to perform one way ANOVA of the obtained data. Means were separated using Duncan multiple range test (DMRT) at *P* ≤ 0.05. Values were presented as means ± SE.

## Results

### Blood Chemistry

The capability of TPJ to rectify the impact of ACR on the level of liver function enzymes as well as some essential blood parameters was tested. TPJ alone did not induce significant changes in all estimated parameters compared with the control group. ACR increased AST (4.7-fold) and ALT (4.2-fold) compared with controls. Co-treatment of TPJ+ACR reduced the high scores of AST and ALT compared with the ACR group (Table 2). Also, ACR reduced the GGT level to almost 0.5-fold compared with the control and TPJ groups while co-treatment of TPJ+ACR highly increased the GGT level to an insignificant level compared with the control and TPJ groups (Table 2). Urea level was increased by ACR to about 3.45-fold compared with the control. The TPJ had a protective effect against ACR by which it reduced the urea level to 1.3-fold of the control group. Albumin and total proteins were reduced by ACR to 0.39-fold and 0.45-fold of the control, consecutively, while the TPJ had a protective effect against the ACR effect by increasing albumin and total proteins to an insignificant level compared with the control and TPJ (Table 2).

**Table 2:** Protective effect of Taify Pomegranate juice (TPJ) on ACR-induced liver dysfunction in rats.

	**Control**	**ACR**	**TPJ**	**TPJ + ACR**
AST (U/l)	31.7 ± 2.16^c^	149.99 ± 10.88^a^	29.77 ± 1.00^c^	61.24 ± 1.73^b^
ALT (U/l)	32.18 ± 1.14^c^	135.12 ± 3.87^a^	31.76 ± 1.79^c^	56.66 ± 2.35^b^
GGT (U/l)	4.14 ± 0.35^b^	1.9 ± 0.10^a^	3.70 ± 0.25^b^	3.9 ± 0.19^b^
Urea (mg/dl)	19.84 ± 1.40^d^	58.10 ± 1.2^a^	21.26 ± 1.78^c^	36.85 ± 1.01^b^
Albumin (g/dl)	6.99 ± 0.33^b^	2.65 ± 0.14^a^	5.73 ± 0.60^b^	6.80 ± 0.53^b^
Total proteins	10.09 ± 0.30^b^	4.85 ± 0.27^a^	9.6 ± 0.50^b^	9.99 ± 0.26^b^
(g/dl)				

*Values are expressed as means ± SE. Means in the same raw with different superscript indicate significant statistical differences at p < 0.05*.

### *In vivo* Antioxidant Capacity of TPJ

ACR induced a significant increase in MDA (3-fold) and NO (3-fold) levels. It also decreased the level of antioxidant enzymes, including catalase (0.4-fold), SOD (0.56-fold), and GSH (0.5-fold) compared with the control (Table 3). TPJ alone reduced the MDA level below its level in the control and increased the SOD and GSH levels compared with the control, whereas it did not have significant changes on the catalase and the NO levels (Table 3). Co-treatment of TPJ+ACR showed a protective effect of the ACR oxidative stress. This was indicated by significant reduction of MDA, increase of catalase, SOD, and GSH, and decrease of NO compared with the ACR group (Table 3).

**Table 3:** Protective effects of Taify pomegranate juice (TPJ) against ACR-induced alterations on serum MDA, catalase, SOD, GSH, and NO levels.

	**MDA** **(nmol/ml)**	**Catalase** **(U/ml)**	**SOD** **(U/ml)**	**GSH** **(U/ml)**	**NO** **(nmol/ml)**
Control	24.41 ± 1.00^c^	3.65 ± 0.22^a^	32.76 ± 0.64^b^	33.45 ± 0.59^b^	22.00 ± 0.75^c^
ACR	72.52.1.77 ± 1.77^a^	1.50 ± 0.06^c^	18.30 ± 0.59^d^	16.72 ± 1.44^d^	65.86 ± 1.78^a^
TPJ	20.17 ± 1.40^d^	3.68 ± 0.29^a^	42.30 ± 1.17^a^	43.12 ± 1.18^a^	25.14 ± 0.71^c^
TPJ+Acylamide	33.41 ± 1.06^b^	2.90 ± 0.25^b^	26.88 ± 0.93^c^	27.29 ± 0.78^c^	40.04 ± 2.38^b^

*Values are expressed as means ± SE. Means in the same column with different superscript indicate significant statistical differences at p < 0.05*.

### Anti-inflammatory Effect of TPJ

TPJ alone enhanced the level of the anti-inflammatory cytokine IL-10 while ACR reduced its level compared with the control. Co-administration of TPJ and ACR abrogated the ACR effect on the IL-10 (Table 4). TPJ reduced the inflammatory cytokine TNF-α compared with the control, whereas ACR significantly induced higher levels of TNF-α compared with the control. Co-administration of TPJ and ACR significantly reduced TNF-α levels compared with the control group (Table 4). Similarly, ACR induced high levels of the inflammatory cytokine IL-6, and PJ alone reduced the IL-6 levels to insignificant state compared with the control. ACR administration with TPJ highly and significantly reduced IL-6 production, indicating that TPJ has an anti-inflammatory effect that was able to rectify the ACR increase of IL-6, TNF-α, and the decrease of the anti-inflammatory IL-10.

**Table 4:** Protective effects of Taify Pomegranate juice (TPJ) against ACR-induced alterations in serum cytokine levels.

**Cytokine**	**Control**	**ACR**	**TPJ**	**TPJ + ACR**
IL-10	118.00 ± 5.60^b^	77.60 ± 3.93^d^	144.40 ± 7.05^a^	102.60 ± 2.50^c^
TNF-α	87.80 ± 1.15^c^	305.20 ± 15.94^a^	92.60 ± 4.91^c^	161.80 ± 5.21^b^
IL-6	77.00 ± 5.92^c^	274.00 ± 11.10^a^	89.20 ± 3.06^c^	138.80 ± 1.07^b^

*Values are expressed as means ± SE. Means in the same row with different superscript indicate significant statistical differences at p < 0.05*.

### TPJ Abrogated the ACR-Induced Suppression of Endogenous Antioxidant Genes SOD and Catalase

**Figure 1:**
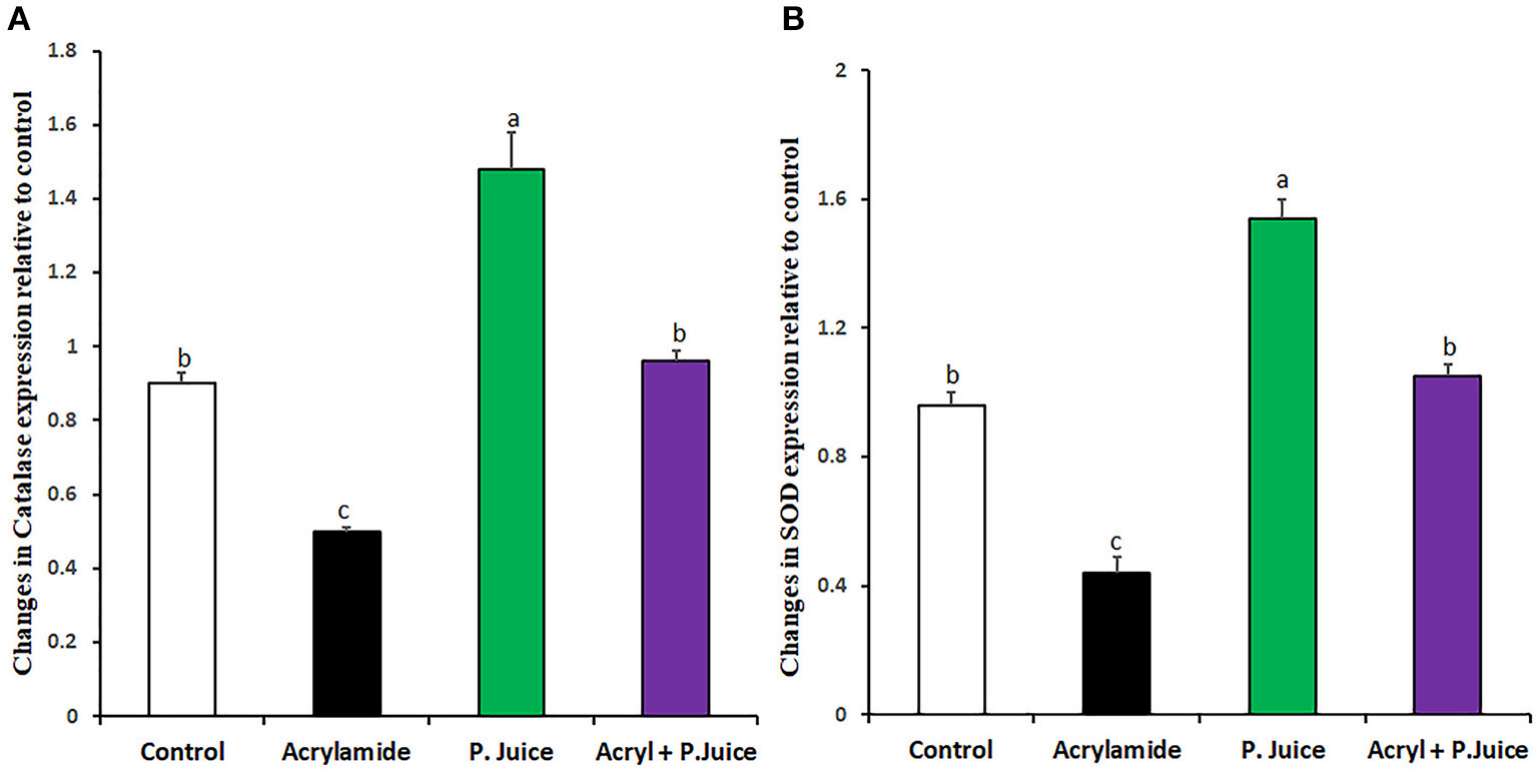
Antioxidant enzyme gene expression in response to ACR and TPJ of Catalase **(A)** and SOD **(B)**. Values are expressed as means ± SE. Means with different letters are significant (*p* < 0.05).

To investigate the capability of TPJ to rectify the ACR-induced oxidative stress, its effect on the expression of the endogenous antioxidative enzymes was investigated. Treatment with ACR reduced catalase expression, whereas treatment with TPJ alone induced its expression compared with the control. On the other hand, co-treatment of TPJ and ACR rectified the catalase expression to a level close to the control and significantly higher than its level in the ACR group (Figure 1A). Also, the expression of SOD was downregulated by ACR while TPJ alone upregulated its expression compared with the control group. Co-treatment of TPJ and ACR had a protective effect of ACR-induced downregulation of SOD compared with the control, where the TPJ was able to rectify the ACR-induced reduction in SOD level (Figure 1B).

### TPJ Rectified the ACR-Downregulation of Nrf2/HO-1 Axis

**Figure 2:**
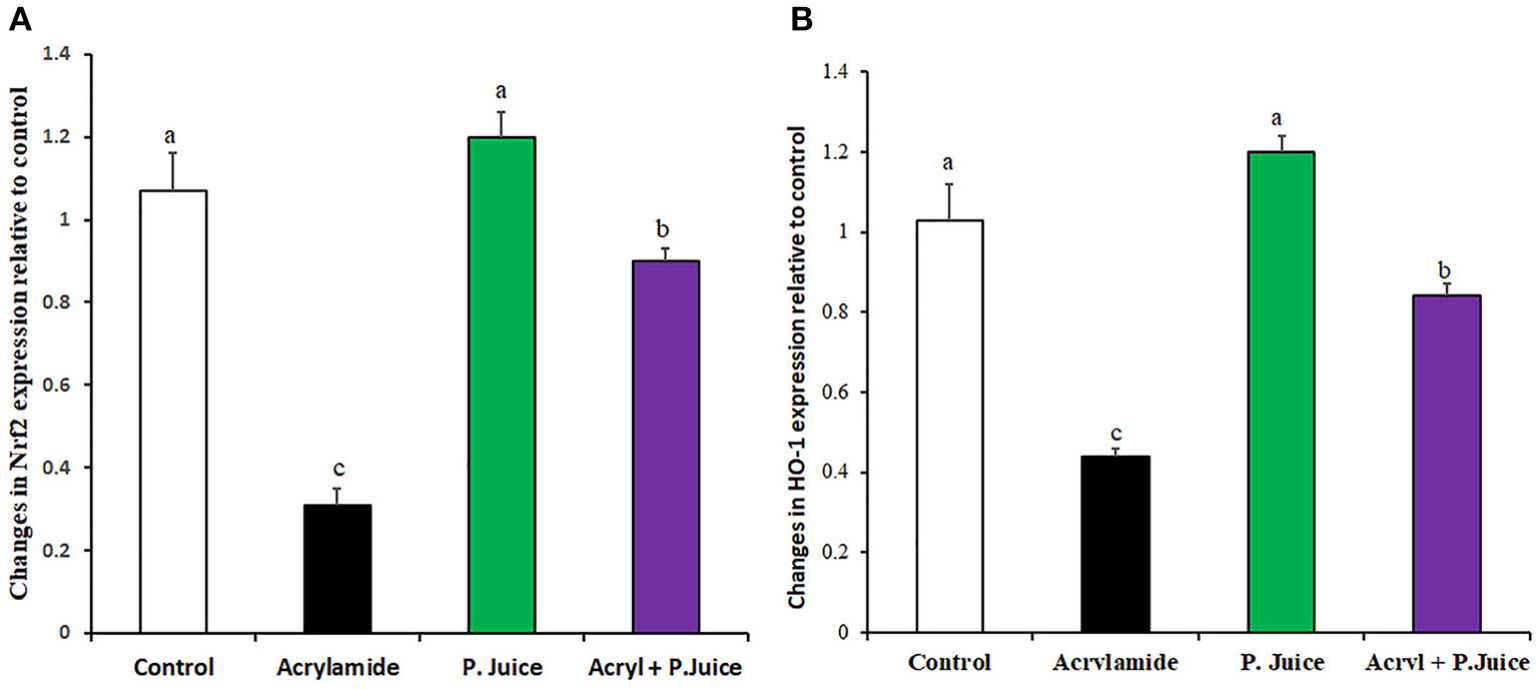
Gene expression of oxidative stress marker genes Nrf2 **(A)** and HO-1 **(B)** in response to ACR and the TPJ in rat liver. Values are expressed as means ± SE. Means with different letters are significant (*p* < 0.05).

ACR and TPJ had diverse effects on the cytoprotective antioxidant marker genes nuclear factor-erythroid 2-related factor 2 (Nrf2) and Heme Oxygenase-1 (HO-1). While ACR greatly suppressed the Nrf2 to 0.31-fold of the control, TPJ enhanced its expression to 1.2-fold compared with the control group. Co-administration of TPJ and ACR significantly normalized the expression of Nrf2 compared with the control or the TPJ groups, yet it was still significantly higher compared with the ACR group (Figure 2A) giving an indication that TPJ had a protective effect from ACR-induced suppression of cytoprotective antioxidant Nrf2 gene. Regarding HO-1 gene, ACR and TPJ had similar expression profile to that of Nrf2. ACR significantly suppressed HO-1 to 0.44-fold of the control; meanwhile, TPJ enhanced its expression level to 1.2-fold of the control. Co-administration of PJ and ACR enhanced its expression to 0.84-fold of the control (Figure 2B), providing evidence that TPJ had a protective effect against ACR-induced oxidative stress.

### Anti-inflammatory Activity of TPJ

**Figure 3:**
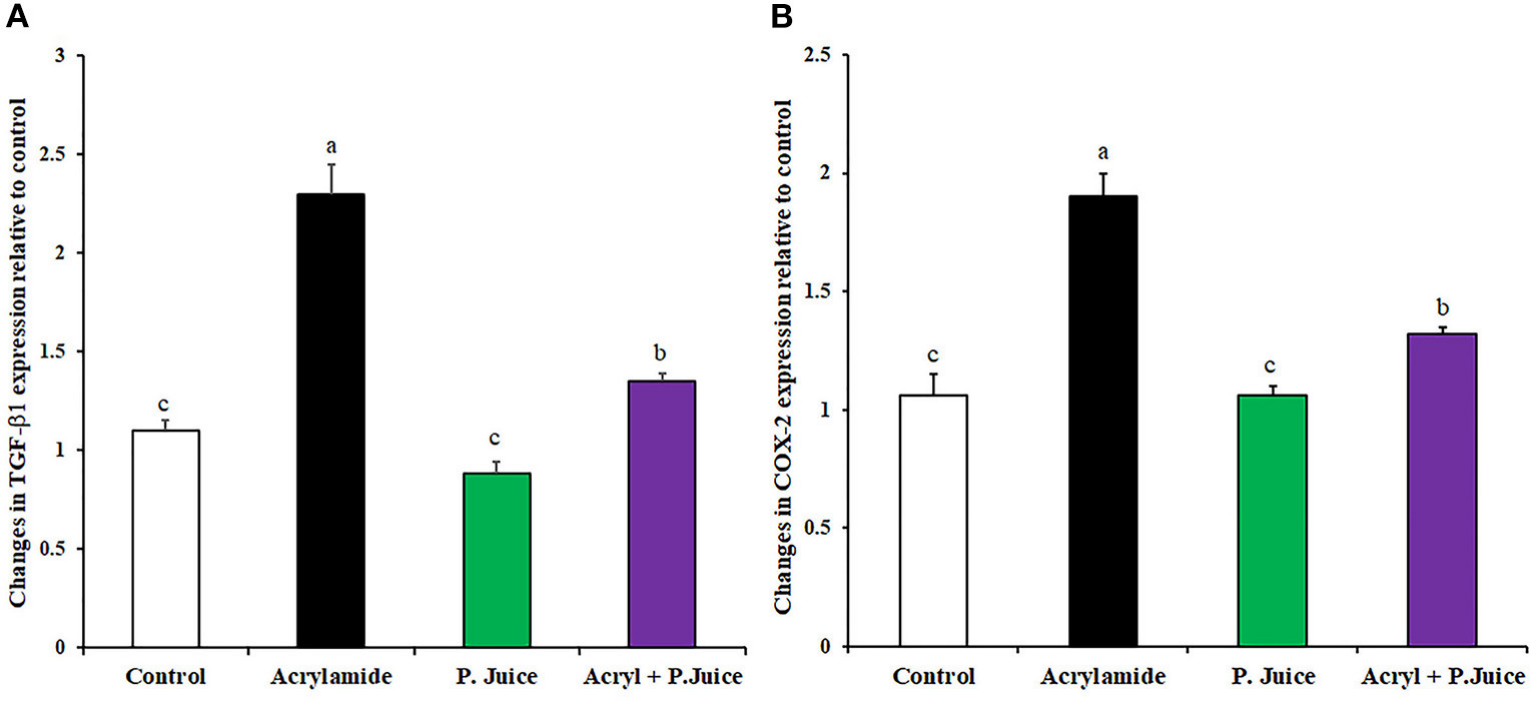
Gene expression of fibrosis-associated genes TGF-B1 **(A)** and COX2 **(B)** in response to ACR and the TPJ in rat liver. Values are expressed as means ± SE. Means with different letters are significant (*p* < 0.05).

The protective effect of TPJ from inflammation was estimated by modulating gene expression of the inflammatory transforming growth factor β1 (TGF-β1) and cyclooxygenase2 (COX2). ACR upregulated the TGF-β1 about 2.3-fold while TPJ downregulated its expression to 0.88-fold of the control. Co-treatment of ACR and TPJ indicated that TPJ abrogated the ACR effect and downregulated the TGF-β1 expression to 1.35-fold of the control, concluding its protective effect (Figure 3A). Treatment with ACR highly induced the expression of the inflammatory COX2 compared with the control with about 1.9-fold compared to the control. TPJ reduced the expression of COX2 to a level close to the control level (1.06-fold). Co-administration of ACR and TPJ reduced the COX2 expression to 1.32-fold of the control, indicating the protective effect of the TPJ (Figure 3B).

### Anti-apoptotic Activity of TPJ

**Figure 4:**
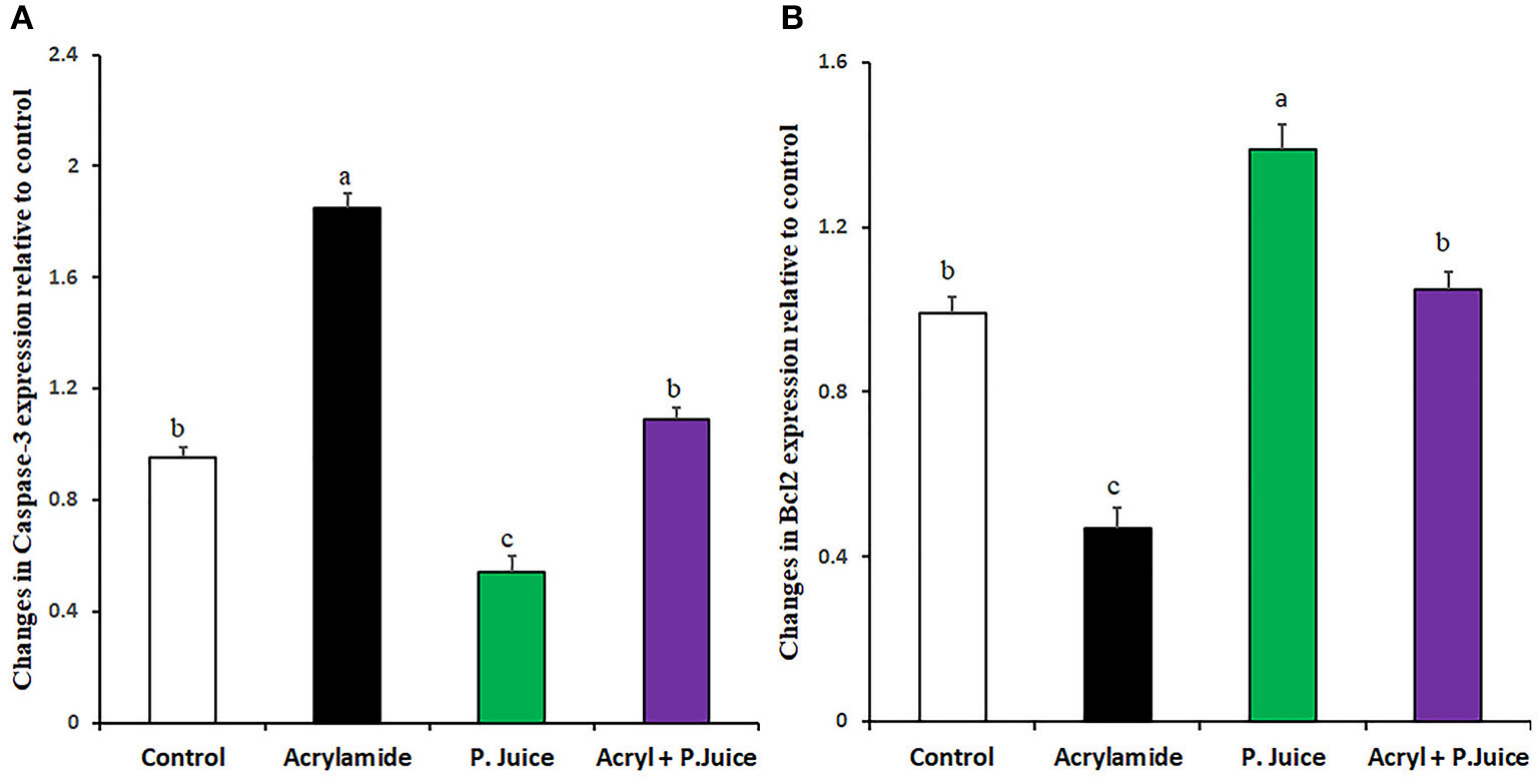
Gene expression of the apoptotic Caspase-3 **(A)** and the antiapoptotic Bcl2 **(B)** genes in response to ACR and the TPJ in rat liver. Values are expressed as means ± SE. Means with different letters are significant (*p* < 0.05).

The apoptotic caspase-3 gene expression was induced by ACR about 1.85-fold, whereas the TPJ significantly reduced its expression to 0.54-fold compared with the control. When TPJ was co-administered with ACR, the TPJ rectified the caspase-3 expression to 1.09-fold of the control (Figure 4A). On the contrary, to the caspase-3 case, ACR was able to suppress the antiapoptotic gene Bcl2 to 0.47-fold while TPJ upregulated the Bcl2 gene 1.39-fold compared with the control. Co-treatment of ACR along with the TPJ showed a protective effect through the enhancement of the Bcl2 gene to a comparable level (1.05-fold) to the control group (Figure 4B).

### Histopathological Protection of TPJ

**Figure 5:**
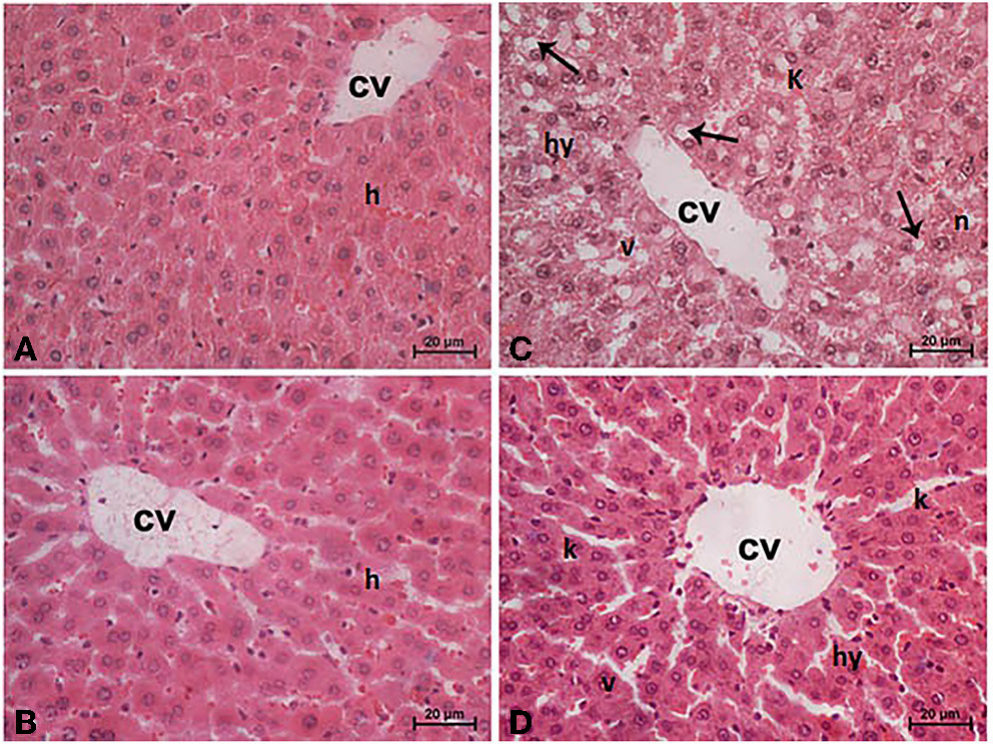
Photomicrographs of liver tissues stained with hematoxylin and eosin in the control and TPJ group **(A,B)**, the acrylamide-treated group **(C)**, and the group co-treated with acrylamide and TPJ **(D)**. Sections from control and TPJ groups **(A,C)** show the liver consisted of CV surrounded by hepatic cords (h). The cords consisted of large hepatocytes with centrally located nuclei and acidophilic cytoplasm. Sections from the acrylamide-treated rats **(B)** the liver tissue showed vacular degeneration (v), hydrobic degeneration (hy). The liver tissues showed proliferation of the vonkupher cells (k). The blood sinusoids showed cellular infiltrates (Table 5). Co-treatment of rats with acrylamide and TPJ **(D)** showed less vacuolar and hydrobic degeneration in some hepatocytes. Some vonkupher cells still proliferated but were not numerous compared with the acrylamide group. Scale bar = 20 μm (original magnification = 200 ×). The morphometric and scoring of hepatic lesions are shown in Table 5.

Sections from normal and TPJ administered rats (Figures 5A,B) displayed normal features of hepatic parenchyma organized in hepatic cords radiating from the central veins (CV) at the center of the hepatic lobule toward the lobular periphery. Sections from acrylamide-treated rats (Figure 5C) showed degenerative changes as indicated from vacuolar hepatocytes (arrows). Sections from acrylamide-treated rats (Figure 5C) showed that vascular degeneration (v) and hydrobic degeneration (hy) of hepatocytes. The liver tissues showed proliferation of the vonkupher cells (k). The blood sinusoids showed cellular infiltrates. Some hepatocyte cells were characterized by necrosis (n). The co-treatment of rats with acrylamide and TPJ (Figure 5D) showed less vacuolar and hydrobic degeneration in some hepatic cells. The Kupffer cells still proliferated but were not numerous compared with the acrylamide group. The detected degenerative changes are illustrated in Table 5. Co-treatment of rats with acrylamide and TPJ (Figure 5D) reduced the degenerative changes and protected rats from the bad effects of ACR. Scale bar = 20 μm (original magnification = 200 ×).

**Table 5:** Ameliorative impacts of TPJ against morphometric analysis and score lesions induced by acrylamide rat's liver.

**Lesion**		**Control**	**ACR**	**TPJ**	**TPJ + ACR**
Frequencies	Inflammatory infiltrate	0^c^	14 ± 2.9^a^	0^c^	2 ± 0.2^b^
	Vacuolar and hydropic degeneration	0^c^	20.0 ± 3.0^a^	0^c^	8.3 ± 2.7^b^
	Von Kupffer cell hyperplasia	0^c^	7 ± 1.3^a^	0^c^	2 ± 0.9^b^
	Single-cell Necrosis	0^c^	15 ± 4.2^a^	0^c^	8 ± 1.6^b^
Areas	Central veins	0.9 ± 0.1^a^	2.9 ± 0. 4^c^	1.1± 0.01^a^	1.2 ± 0.2^b^
	Portal blood vessels	1.1 ± 0. 3^a^	5.1 ± 0.7^c^	2.1 ± 0.2^a^	2.5 ± 0.2^b^
	Sinusoidal spaces	5.1 ± 0.1^a^	8.1 ± 1.1^c^	4.1 ± 0.3^a^	5.5 ± 1.0^b^

*Values are mean ± SE for 5 slides/group. Means within the same row with different superscripts are significantly different at p < 0.05*.

**Figure 6:**
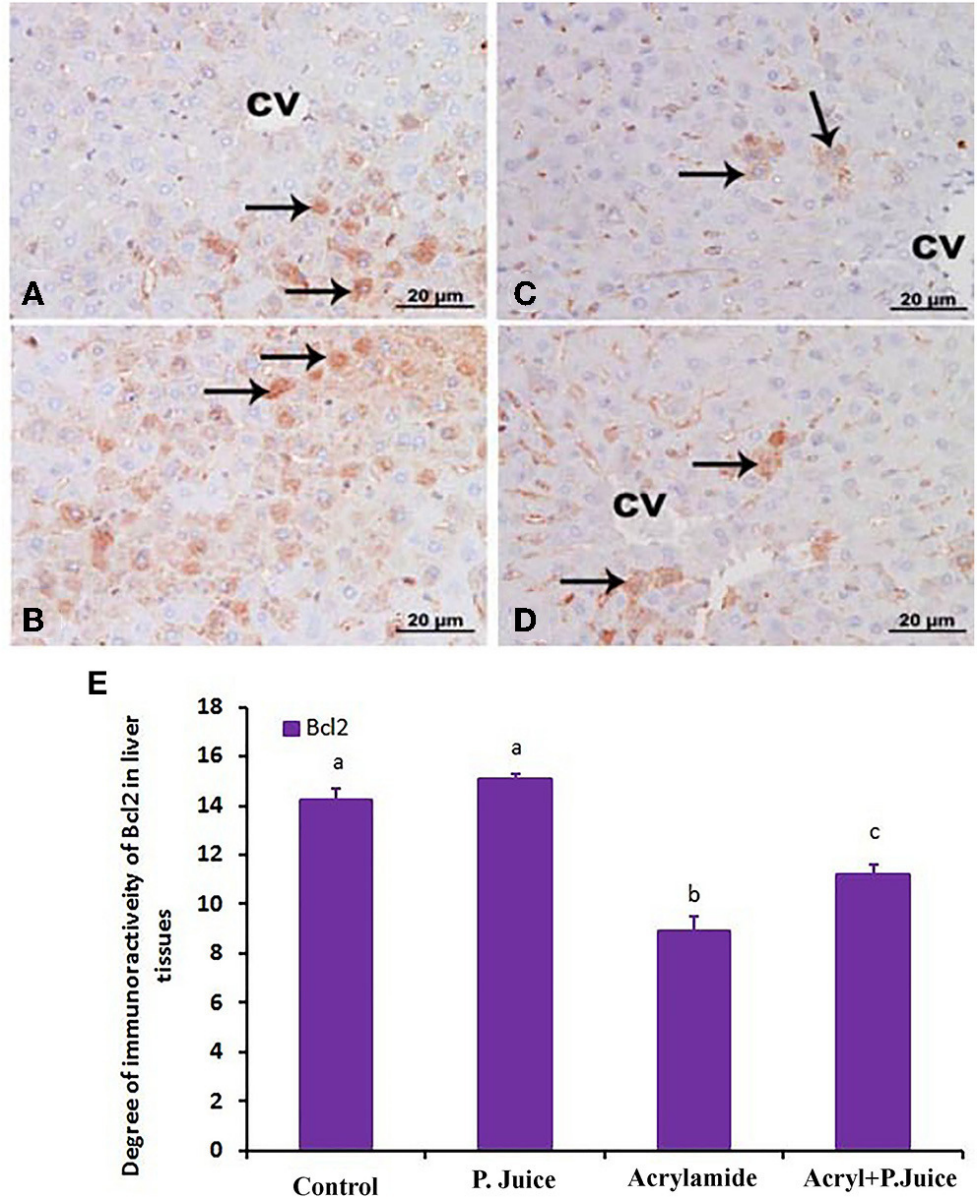
Photomicrographs of hepatic sections immunostained with Bcl2 antibody in the control group **(A)**, the TPJ group **(B)**, the acrylamide-treated group **(C)**, and the group co-treated with TPJ and acrylamide **(D)**. The intensity of immunostaining was remarkable in the control and TPJ-treated groups **(A,B)**, less in the acrylamide-treated group **(C)**, and ameliorated and restored in the co-treated rats **(D)**. Reactive liver cells (arrows) are frequently seen next to CVs. Scale bar = 20 μm (original magnification = 200 ×). The degree of positive immunoreactivity for Bcl2 is graphed in **(E)**. Densitometric values are statistically significant at *p < 0.05 vs. the control and TPJ-treated groups; ^#^*p* < 0.05 vs. the acrylamide-administered group. In panel E, means with different letters indicate significance (*p* < 0.05).

The intensity of Bcl2 immunostaining was remarkable in control (Figure 6A) and TPJ (Figure 6B) as indicated from the strongly positive hepatocytes (arrows), reduced in the acrylamide group (Figure 6C) and partial restored to fair levels in pretreated rats (Figure 6D). The reactive liver cells (arrows) are usually next to CV. Reactive hepatocytes are marked with arrows. Scale bar = 20 μm (original magnification = 400 ×). This immunostaining of Bcl2 is shown in the form of densitometric score and is correlated with the estimated immunoreactivity (Figure 6E) of immunostained liver sections (Figures 6A–D), confirming the protective effect of TPJ.

## Discussion

Plant-derived phytochemicals collectively known as nutraceuticals have biological functions for protection against chronic disease, improving health, delaying aging, or supporting various functions of the body (99). They have gained increasing interest for their nutritional, pharmaceutical, safety, and protective roles against oxidative stress-related metabolic disorders, including allergy, cardiovascular diseases, cancer, diabetes, inflammation, and obesity (99). It has been established that nutraceuticals exert their biological functions through their content of bioactive compounds, mainly polyphenols and flavonoids (100). Besides their direct scavenging activity of ROS, they also have the capability to inhibit ROS production by neutrophils and the inhibition inducible-nitric oxide synthases (iNOS) (100). Pomegranate is consumed fresh or used in cosmetic and nutraceutical products because of its antioxidant, anti-inflammatory, anti-microbial, and antiproliferative attributes, which is based on its content of functional phytochemicals (101).

Our results show that TPJ was able to decrease the ACR-induced increase of AST, ALT, GGT, urea, and total protein when co-administered with ACR. Our results agree with the results obtained from other several studies. PJ showed a protective effect from non-alcoholic fatty liver disease (NAFLD) induced by a high fat diet via lowering the level of AST, ALT, insulin, triglycerides, and glucose compared with the control (102). Also, in NAFLD, PJ decreased the liver enzymes AST and ALT while it increased the antioxidant capacity (103). In addition, similar results were observed using plant-derived products. *Trigonella foenum*-graecum seed oil showed a protective effect against ACR toxicity. While ACR increased serum levels AST, ALT, GGT, and urea, *Tigonella* seed oil supplementation rectified the changed serum parameters and enhanced the antioxidant capacity in the hepatic cells, indicating its protective effects against ACR-induced oxidative stress (43). Furthermore, previous reports show that some PJ active constituents had consistent activity against ACR-induced oxidative stress and its associated metabolic alterations. For example, administration of 50 mg/kg bw quercetin had a protective effect against ACR-induced toxicity through the reduction of oxidative stress and protection from mitochondrial dysfunction (35, 37). Ellagic acid showed protection from ACR-induced neurotoxicity (13). Myricetin had a counteracting activity of ACR-induced oxidative stress through the inhibition of the MEK/ERK signaling pathway (104).

In the current study, ACR increased urea and decreased albumin and total proteins, and TPJ normalized these serum parameters. This aligned with other reported biological activities of PJ. It presented protective effects against sodium fluoride oxidative damages in liver tissue and erythrocytes of rats. It showed an ameliorative effect on hematological parameters, rectified the total protein, albumin, bilirubin levels, and the activities of hepatic marker enzymes (105).

The increase of MDA and NO as well as the decrease in catalase, SOD, and GSH in the current study is in line with the results obtained from previous studies. PJ had protective effect against ACR-induced oxidative stress through the reduction of MDA with increased antioxidant capacity in NAFLD patients (103). Also, PJ compromised the higher levels of lead acetate–induced MDA and GSH, indicating a protective role against lead acetate–induced oxidative stress (106). Similarly, PJ presented a protective effect against the anxiety and depression induced by aluminum trichloride exposure through the enhancement of catalase, SOD, GST, and GSH (107). In addition, PJ showed antifibrotic activity against NDEA-induced liver fibrosis via increasing SOD, GST, and catalase levels (108) and enhanced the antioxidant enzymes, including SOD, catalase, and the GSH levels in liver, kidney, and testis tissues against lead-induced oxidative stress (109). PJ had a protective effect of diethylnitrosamine and phenobarbital-induced hepatic damage by reducing the level of MDA, glutathione reductase (GSR), decreased SOD and GST that were altered by diethylnitrosamine and phenobarbital (110). Prolonged ingestion of PJ alleviated the impact of systemic oxidative stress in mice by lowering oxidative biomarkers including catalase, SOD, GSH, and GSSG (111). High altitude-induced hypoxia and its associated metabolic alterations were protected by PJ via lowering the level of lipid peroxidation in muscle, rectifying the GSH:GSSG ratio (112).

Endogenous antioxidant enzymes, including SOD, catalase, and glutathione peroxidase are induced by oxidative stress through the binding of nuclear factor-erythroid 2-related factor 2 (Nrf2) transcription factor to the antioxidant responsive elements (ARE) in their promoter regions (100, 113). Nrf2 regulates ARE-regulated genes (113, 114). Normally, Nrf2 is bound to kelch-like protein-1 (KEAP1) and localized in the cytoplasm and under oxidative stress, and Nrf2 is released and translocated to the nucleus. In the nucleus, it induces the transcription of antioxidant enzymes and provokes the transcription of HO-1 expression. Also, oxidative stress is the stimulant of nuclear factor-kappa B (NF-κB), inflammatory cytokines, such as interleukin (IL)-6, IL-17, and tumor necrosis factor-alpha (TNF-α) (115). TNF-α is an inflammatory cytokine that is responsible for a wide range of molecular and signaling pathways underlying necrosis and/or apoptosis. It also plays an indispensable role in cancer and infection resistance (116).

ACR downregulated the antioxidant activating genes Nrf2, HO-1; upregulated the inflammatory genes TGF-1β, and COX2; induced the inflammatory cytokines IL-6 and TNF-α, and reduced the anti-inflammatory cytokines IL-10. On the other hand, preadministration of TPJ rectified these inflammatory and anti-inflammatory parameters. This provides strong evidence that TPJ exerts an anti-inflammatory activity against ACR-induced oxidative and inflammatory stress. Several previous studies report results in line with ours. CuO-NPs-induced oxidative stress was compromised using PJ via the downregulation of the Nrf2/HO-1 axis (117). In addition, PJ downregulated the expression of COX2 and Nrf2 induced by NDEA in fibrotic rat liver (108). *Trigonella foenum-graecum* seed oil reduced the ACR-elevated levels of inflammatory cytokines, including IL-1β, IL-6, and TNF-α (43).

TPJ normalized the altered expression profile of caspase-3 and Bcl2 genes altered by ACR, which agrees with previous published studies. PJ downregulated the caspase-3 induced by diethylnitrosamine and phenobarbital (110) and downregulated the CuO-NPs-induced high expression of caspase-3 (117). Garlic and *Spirulina maxima* had a protective effect from Pb-induced neurotoxicity, including the proapoptotic associated increase of caspase-3 expression as well as the altered acetyl cholinesterase enzyme activity and oxidative stress parameters (118).

At the histopathology level, TPJ protected hepatic tissue from the ACR-induced histopathological effects. Similar activities of PJ are reported by other authors. PJ reduced the histological changes associated with non-alcoholic fatty liver disease, including disturbed hepatic architecture, dilatation and congestion of central veins, blood sinusoids and portal veins, cytoplasmic vacuolation, mitochondrial structural changes, dilatation of endoplasmic reticulum in addition to nuclear structural changes like condensed chromatin, irregular shrunken nuclei and vacuolated nuclei, inflammatory cellular infiltrations, deposition of collagen fibers around the central vein, blood sinusoids, portal areas and in between the hepatocytes in addition to significant increase in number of hepatic stellate cells that was proved by electron microscope and confirmed by immunohistochemical study (119). Also, PJ was able to protect tissue damage induced by lead acetate in kidney, liver, and heart as well as reduced the accumulation of lead in kidney and testis (109). In addition, PJ had a significant ameliorative effect against cisplatin-induced renal damage at the histopathological level, giving evidence that PJ could be used as dietary supplement for individuals during chemotherapy treatments (120). Moreover, PJ reduce the NAFLD-associated hepatic steatosis, ballooning, lobular inflammation, and portal inflammation (102).

The obtained results indicate that TPJ had an integrated protection effect against ACR at various levels, including serum parameters, gene expression in tissues, and histological parameters. This integrated protection of PJ was observed in other previous studies (90, 106, 109).

The represented biological activities of TPJ, including antioxidative, anti-inflammatory, and antiapoptotic activities could be attributed to its components of various compounds with previously shown beneficial biological activities. Also, prepared from plants growing at high altitude, the TPJ has some unique components, such as syringic acid, quercetin, naringenin, and myricetin, that might have contributed to its biological activities reported in the current study. In conclusion, TPJ has the potential to ameliorate the toxicity of acrylamide through the regulation of anti-inflammatory, anti-apoptotic, and antioxidant molecular pathways.

## Data Availability Statement

The raw data supporting the conclusions of this article will be made available by the authors, without undue reservation.

## Ethics Statement

The animal study was reviewed and approved by Ethical committee, Taif University.

## Author Contributions

All authors listed have made a substantial, direct, and intellectual contribution to the work and approved it for publication.

## Funding

This study was supported by the Deputyship for Research and Innovation, Ministry of Education, in Saudi Arabia under project number 1-441-128.

## Conflict of Interest

The authors declare that the research was conducted in the absence of any commercial or financial relationships that could be construed as a potential conflict of interest.

## Publisher's Note

All claims expressed in this article are solely those of the authors and do not necessarily represent those of their affiliated organizations, or those of the publisher, the editors and the reviewers. Any product that may be evaluated in this article, or claim that may be made by its manufacturer, is not guaranteed or endorsed by the publisher.
